# Physicochemical and Sensory Characteristics of a Chagalapoli Fruit (*Ardisia compressa*) Beverage Fermented Using *Saccharomyces cerevisiae*

**DOI:** 10.1155/2019/9687281

**Published:** 2019-10-13

**Authors:** Ana Flores-García, Rubén Márquez-Meléndez, Erika Salas, Guillermo Ayala-Soto, Iván Salmerón, León Hernández-Ochoa

**Affiliations:** Universidad Autónoma de Chihuahua, Facultad de Ciencias Químicas, Departamento de Posgrado, Circuito Universitario, Campus Universitario #2, Chihuahua C.P. 31125, Mexico

## Abstract

Chagalapoli fruit (*Ardisia compressa*) is similar to *Vaccinium myrtillus *(berries) with high-polyphenol content. The objective of this study was to evaluate the physicochemical properties of Chagalapoli fruit and to determine the conditions for the preparation of a fermented beverage using *Saccharomyces cerevisiae *yeast, evaluating the impact on sensory properties. The fermentation process lasted 4 days at 27°C, with absence of light and a fixed pH of 3.8. The phenolic contents obtained in samples were 1.27 epicatechin mg/mL in filtered juice, 1.59 epichatechin mg/mL in filtered fermented beverage, 1.91 epichatechin mg/mL in partially filtered juice and 3.19 epichatechin mg/mL in partially filtered fermented beverage. An affective test was carried out to determine the sensory acceptability of the final product, evaluating the flavor, color and aroma parameters. The fermented beverage with the greatest preference on color and flavor attributes was the partially filtered fermented beverage.

## 1. Introduction

Chagalapoli (*Ardisia compressa* subsp. *Myrsinaceae*) belongs to the *Myrsinaceae *family, an extensive family of trees with approximately 500 species [1]. The chagalapoli fruit has a round shape of approximately 12 mm in diameter, with a purple red color that turns black once it reaches maturity; its shell is smooth and delicate, its seed represents up to 50% of its total weight, and the ripe fruit has a bittersweet, mildly astringent taste [2]. In Mexico it is distributed in the states of Chihuahua, Chiapas, San Luis Potosí, Tlaxcala, Hidalgo, Aguascalientes, Puebla, Tamaulipas and Veracruz [3]. Chagalapoli fruit has a similar composition to common berries with a high phenolic content; that is the reason for the high interest in this fruit from a health point of view [4]. The fruit is used to make food products such as juices, jams, and liqueurs, considered to possess antioxidant and antimicrobial properties. In the state of Veracruz the fruit is used against digestive diseases [5]. Chandra and Mejia [6] quantified the total polyphenols of the *Ardisia compressa* leaf extract, obtaining values of 0.58 mg of gallic acid equivalent per mL mgGAE/mL DL. They identified the presence of gallic acid, catechin, epicatechin gallate, ardisin and kaempferol. Heredia-Vasquez [3] determined the total polyphenol content of chagalapoli fruit and obtained 1.74 mgGAE/mL in *Ardisia compressa kunth*; Joaquín-Cruz et al. [4] obtained 1.051 ± 0.43 mgEAG/g and identified the polyphenols of flavonols, flavan-3-ols (catechin and proantocyanidin dimers) and hydroxycinnamoyl derivatives. Jácome-Hernandez [2] obtained 1,638.12 ± 74.98 mgGAE/kg in dry weight (DW). Fermented beverages, such as grape wines, have been associated with health benefits due to the presence of large amounts of phenolic compounds, which are claimed to possess antioxidant properties and have an important role in the prevention of deleterious processes such as ageing, diabetes, cancer, neurological disorders, atherosclerosis and cardiovascular diseases [7]. Moreover, several studies have shown that these beverages induce relaxation in isolated vessels, which makes them important allies for cardiovascular protection [8]. This means that it is necessary to carry out research studies focused on the physicochemical characterization of the fruit and the process of making a fermented beverage that will promote the potential of the chagalapoli fruit. For this reason, the aim of this work was to evaluate the physicochemical properties of the chagalapoli (*Ardisia compressa*) fruit and determine the conditions for the preparation of a fermented beverage using *Saccharomyces cerevisiae*, evaluating the impact on the physicochemical and sensory properties of the final product.

## 2. Material and Methods

### 2.1. Materials

Acetonitrile and formic acid were high-performance liquid chromatography grade. Glucose, Epicatechin, Kaempferol, Quercetin, Catechin, Chlorogenic and Folin-Ciocalteu reagents were provided by Sigma-Aldrich. Milli-Q water was produced using an Elix Millipore water purification system.

### 2.2. Raw Material

Chagalapoli (*Ardisia compressa*) fruit was collected in Aguascalientes, Mexico. 20 kg were manually selected and washed with chlorine (0.05 mL/L). Samples were stored at 4°C for further analysis.

### 2.3. Characterization of Raw Material

Proximal analysis of chagalapoli (*Ardisia compressa*) was determined according to the AOAC methods; protein (976.05) determined by Micro kjeldahl (Nx6.25), moisture (934.06), ash (942.05), fat (920.39), crude fiber (962.09) and carbohydrates.

### 2.4. Must Preparation

Chagalapoli fruit was extracted using a destoner machine (Bertuzzi, Brugherio, Milan, Italy) with a 0.2 sieve separating the seed. The experiment was divided into two batches: Filtered juice (all crushed fruit was filtered), and partially filtered juice (75% filtered crushed fruit and 25% unfiltered fruit). The filtration consisted in separating residues from the juice by centrifugation. Must was prepared according to the methods of Dias et al. [9], with minor modifications. Tartaric acid was added to adjust pH to 3.8 in order to inhibit bacterial growth, and sulfur dioxide was added in the form of potassium metabilsulfite at concentrations of 100 mg/L.

### 2.5. Yeast Strains

Commercial *Saccharomyces cerevisiae* yeast was used and the inoculum was done following the recommendations of the manufacturer (Red Star brand, provided by Maltas e Insumos Cerveceros S.A. de C.V.). 0.173 g of dried yeast was added to 173 mL of warm water with 1.73 g of sucrose at 35–38°C for 20 min, it was left to cool for 10 min. Initial cell density of yeast was 1.9 × 10^6^ ± 0.2 cells/mL.

### 2.6. Fermentation Conditions

Once the must preparation is done according to 2.4 and yeast inoculum is done according to 2.5 were intro out in a 1L bioreactor (BioBundle, Applikon Biotechnology, Netherlands) at 27°C. Fermentation process was evaluated every 24 h and considered complete when the specific gravity was stable. At the end of the fermentation, the beverage was transferred to a glass bottle and stored at 10°C. After 24 h, the beverage was transferred to a new bottle and after 10 days the beverage was filtered and stored at 5°C in 350 mL glass bottles filled to the top to avoid oxygen entrance [11].

### 2.7. Analysis of Juice and Fermented Beverage

Titratable acidity (TAC), specific gravity, pH, cell counts (Neubauer chamber, Celeromics France) total soluble solids (TSS) were determined according to García and Xirau [12]. Sulfur dioxide, volatile acidity (VA) and alcoholic strength by volume were determined after fermentation and storage using methods of the International Organisation of Vine and Wine [13]. Colorimetric properties were measured in terms of CIELAB parameters using a colorimeter model CR400 (Konica Minolta sensing NJ USA) and expressed in terms of rectangular (*L*^∗^, *a*^∗^, *b*^∗^) color coordinates. *L*^∗^ indicates lightness ranging from black (0) to white (100). *a*^∗^ value ranges from red to green and *b*^∗^ ranges from yellow to blue.

### 2.8. Determination of Reducing Sugar

The reducing sugar was obtained using the method of Miller [14]. Briefly, 0.5 mL of sample was added with 0.5 mL of 3,5-dinitrosalicylic acid (DNS) reagent, kept in a boiling water bath for 5 minutes, then the reaction was stopped by placing the test tubes in a cold water bath, 5 mL of distilled water was added and it was left to rest for 15 minutes. Absorbance was determined at 540 nm in an Absorbance microplate reader model EL × 808 (Biotek VT USA). All the experiments were performed in triplicate. The reducing sugar content was calculated based on a standard glucose curve.

### 2.9. Determination of Total Sugar

The total sugar content was determined using the method of Dubois [15] and Chow and Landhausser [16]. 2 mL of sample was diluted in 2 mL of 5% phenol solution; it was boiled in a water bath for 5 min and then cooled with ice. Then 5 mL of H_2_SO_4_ was added and it was stirred, then it was left to rest during 30 min. Absorbance was determined at 490 nm in an Absorbance microplate reader. All experiments were performed in triplicate.

### 2.10. Total Phenolic Content

The total phenolic content was determined using the method of Folin-Ciocalteu [17]. 30 *µ*L of sample was diluted in 3 mL of water and 200 *µ*L of Folin-Ciocalteu reagent and 600 *µ*L of a sodium carbonate solution was added. Afterwards this sample was warmed up at 40°C for 20 min, then it was cooled to room temperature and after 15 min the absorbance was measured at 760 nm. Total phenolic content was calculated as gallic acid equivalent based on a standard gallic acid curve. All the experiments were performed in triplicate.

### 2.11. HPLC-DAD Analysis

Samples were filtered through a 0.45 *µ*m membrane filter (Millipore Corporation). Samples were analyzed on an Agilent 1100 HPLC (Agilent, Technologies CA USA) equipped with a diode detector, with wavelengths at 280 nm, 320 nm and 360 nm. A C18 column (Phenomenex) was used for the stationary phase. The mobile phase consisted of two solvents: (A) water/formic acid (99:1; v/v), and (B) acetonitrile (100%). The gradient employed was: isocratic 0% B for 8 min, 10% B for 2 min, 20% B for 13 min, 30% B for 7 min, 40% B for 15 min, 80% B for 5 min, 100% B for 5 min. Flow rate was set at 1 mL/min and a temperature of 25°C. Phenolic identities were assigned based on their retention characteristics and UV-visible spectra. For the identification and quantification of total phenolics compounds external standard calibration curves of the reported compounds were used (Epicatechin, Kaempferol, Quercetin, Catechin, Chlorogenic).

### 2.12. Sensory Analysis

The final beverages were evaluated by a panel of 32 adult men and women. 20 mL samples at 10°C were given in transparent glasses marked with three-digit random numbers. Fermented beverages were evaluated by acceptability for appearance (color), aroma and flavor according to a 5-point hedonic scale were 0 correspond to least appreciated (dislike extremely) and 5 correspond to most appreciated (like extremely).

### 2.13. Statistical Analysis

Data for all of the measurements were obtained in triplicate and expressed as mean ± standard deviation. Statistical analyses during fermentation (pH, relative density, total soluble solids (TSS) and total titratable acidity (TAC)) were performed with a one-way analysis of variance and means comparison (Tukey). Fermented beverages (filtered and partially filtered beverages) were compared with the filtered and partially filtered juices by means of a *t*-test to determine the significant difference between samples. A significance level *p* < 0.05 was adopted. Different letters were used to label significantly different values. This statistical treatment was carried out using Minitab 17 Statistical Software.

## 3. Results and Discussion

### 3.1. Physicochemical Characteristics of Ardisia compressa

Results obtained in the proximal composition are shown in Table 1. The water content of the fruit was 80.5%, similar to values reported by Joaquin-Cruz et al. [4] who reported 86.8% in *Ardisia compressa* from Veracruz. The Chagalapoli fruit used in this research had a pH level of 4.2 ± 0.1. This result was higher than the values reported by Jacome-Hernandez [2] (pH 2.91) and Joaquin-Cruz et al. [4] (pH 2.73). Likewise, total soluble solids were different from those reported by Jacome-Hernandez [2] (10.5%), while our results were 17.7 ± 0.05%. This could be attributed to the fact that each fruit comes from different locations, weather conditions, harvest season, storage, etc. that affect the characteristics of each fruit. Low pH could contribute to a decrease of general sensory quality of the beverage to an unacceptable level [18].

### 3.2. Analysis during Fermentation

Table 2 shows the results obtained of pH, specific gravity, total soluble solids (TSS) and total titratable acidity (TAC). It was observed that pH values of the beverages did not have a significant difference during the fermentation process, in fermented filtered chagalapoli the change was from 3.84 ± 0.04 to 3.62 ± 0.09 and in fermented partially filtered it was from 3.87 ± 0.2 to 3.54 ± 0.1. The TAC did not exhibit significant difference in either of the batches (8.9 g/L to 8.6 g/L for the filtered sample and 8.9 g/L to 9.2 g/L tartaric acid for the partially filtered sample); in fermented fruit the limit values are 5.5 g/L to 9 g/L (Norma Oficial Mexicana PROY-NOM-199-SCFI-2015) which indicates that TAC obtained in the filtered sample is within the permitted limits. Specific gravity showed significant difference between samples, which showed a tendency to decrease with time; batches had the same value at the end of fermentation. During alcoholic fermentation of both batches of *Ardisia compressa*, initial value of yeast was 1.9 × 10^6^ ± 0.2 cells/mL. Number of microorganisms increases due to environmental conditions and sugar content decreases rapidly. Maximum value of yeasts was obtained on the second day with an amount with density of 4.1 × 10^7^ ± 0.1 cells/mL; this is relevant because it suggests that at this point the yeast was in optimal conditions that favor the production of alcohol. A decrease of cell density was observed after the third day and fermentation concluded without a significant difference on the fourth day with a value of 4.4 × 10^6^ ± 0.2 cells/mL.

### 3.3. Color Parameters

Table 3 shows CIELAB color parameters obtained. *L*^∗^ value was 27.5–30.34 in the filtered sample and 30.75–31.09 in the partially filtered sample. It was observed that values increased during fermentation, which indicates the samples are darker. *a*^∗^ obtained in filtered samples was 8.87–8.42 and in partially filtered samples was 7.15–6.92. It was observed that values decreased during fermentation which indicates a red tone. *b*^∗^ values was 14.50–17.50 in filtered samples and 16.59–16.28 in partially filtered samples, which indicates they *b*^∗^ values increased in the filtered samples and decreased in partially filtered samples, obtaining a yellow color. Angle of hue and chromaticity defined the sample within the red color, presenting significant differences in all samples. Heredia-Vasquez [3] reported different values in *L*^∗^, *a*^∗^ and *b*^∗^, obtained low luminosity and more tendency towards red color. Differences can be justified by the type of fruit used as well as by climatic condition, region, harvest time, etc.

### 3.4. Analysis of Fermented Beverage

Fermented filtered and partially filtered beverage of chagalapoli (*Ardisia compressa*) fruit obtained a similar result in alcoholic degree method, 6% and 6.4% respectively. Official Mexican Standard NOM-199-SCFI-2017 specifies the limit values for fermented fruit beverages from 6% to 12%; the values obtained from chagalapoli (*Ardisia compressa*) fermented beverage are within the established range. Chowdhury and Ray [19] reported a 6% of alcohol content in a fermented jamun berry (*Syzygium cumini L.*) beverage.

In relation to volatile acidity 0.32 g/L was the value obtained for filtered fermented beverage and 0.36 g/L for partially filtered beverage; Mena et al. [10] determined the volatile acidity of fermented beverages of 3 pomegranate varieties, for which they obtained values of 0.33, 0.36, and 0.26 g/L. These results obtained suggest that the sugar consumption produce different acids (citric, malic, acetic and tartaric acids).

The Official Mexican Standard NOM-199-SCFI-2017 allows a maximum 50 mg/L of free sulfur dioxide and 350 mg/L for total sulfur dioxide. To determine the sulfur dioxide in fermented beverages the free sulfur dioxide was calculated first; 17.06 ± 0.73 mg/L was determined for free sulfur dioxide and 18.77 ± 4.8 mg/L for total sulfur dioxide. For the total sulfur dioxide there was no significant difference between the samples, 57.6 ± 3.3 and 58.88 ± 3.8 were obtained, under the standard the samples are within the permitted limits. Oliveira et al. [11] reported similar results in cagaita wines (*Eugenia dysenterica DC*), they obtained from 22 mg/mL to 12 mg/L of total sulfur dioxide. Kelebek et al. [21] reported 8.2 ± 0.08 mg/L of free sulfur dioxide and 73.3 ± 0.12 mg/mL of total sulfur dioxide in orange wine.

### 3.5. Reducing Sugar

Results are shown in Table 4. The values for filtered juice and partially filtered juice were 132.08 ± 1 and 96.63 ± 2 mg/mL, respectively. For filtered and partially filtered fermented beverage, the values decreased to 3.42 ± 0.3 mg/mL and 3.58 ± 0.3 mg/mL, respectively. Similar results were reported by Oliveira et al. [11] where values of 1.2 mg/mL and 2.4 mg/mL of reducing sugars were obtained in Cagaita fruit wine.

### 3.6. Total Sugar Contents

Total sugar contents results are shown in Table 4. Filtered juice had more total sugars than partially filtered juice with 137.83 ± 0.9 mg/mL and 83.04 ± 0.6 mg/mL, respectively. These results may be compared to the report by Kosseva et al. [20] where blueberry presented values of 155.2–164.7 g/kg, while in red grapes it was around 200 g/kg or more at maturity. Comparing these results with reported values of blueberry, chagalapoli (*Ardisia compressa*) sugars are lower. There was a reduction during the fermentation, and at the end of samples 3.74 ± 0.1 mg/mL and 3.27 ± 0.03 mg/mL of total sugars were obtained in final products of fermented beverages. Kelebek et al. [21] reported 120.19 mg/mL in orange juice and 48 mg/mL in fermented orange juice. These results could differ since the orange is considered a citrus fruit and because the sugars present in each fruit are different. Since the methods for reducing and total sugars are different, results are not considered complementary.

The results obtained for total sugar content and reducing sugar content in the partially filtered samples showed the limitation of sugar desimentation, this may explain the variation in the results obtained.

### 3.7. Analysis of Phenolic Compounds

#### 3.7.1. Folin-Ciocalteu Method

The total polyphenol content by the Folin-Ciocalteu method in chagalapoli (*Ardisia compressa*) juices was 1.8 ± 0.03 mgGAE/mL for filtered juice and 1.75 ± 0.1 mgGAE/mL for partially filtered juice. Similar values were previously reported by Heredia-Vásquez [3], who obtained values of 1.74 mgGAE/mL in *Ardisia compressa kunth*; Joaquín-Cruz, et al. [4] obtained 1.051 ± 43.5 mgGAE/g in chagalapoli fruit. In filtered and partially filtered fermented beverage the values decreased to 1.11 ± 0.02 mgGAE/g and 1.59 ± 0.19 mgGAE/g, respectively. Martins de Sá et al. [7] reported 1.105 ± 57 mgGAE/mL in a fermented jabuticaba (*Myrciaria jaboticaba*) beverage. Johnson et al. [22] reported a total polyphenol content of 0.3754–0.6571 mgGAE/mL in blackberry (*Vaccinium* spp.) fermented beverage.

#### 3.7.2. HPLC-DAD Analysis

**Figure 1:**
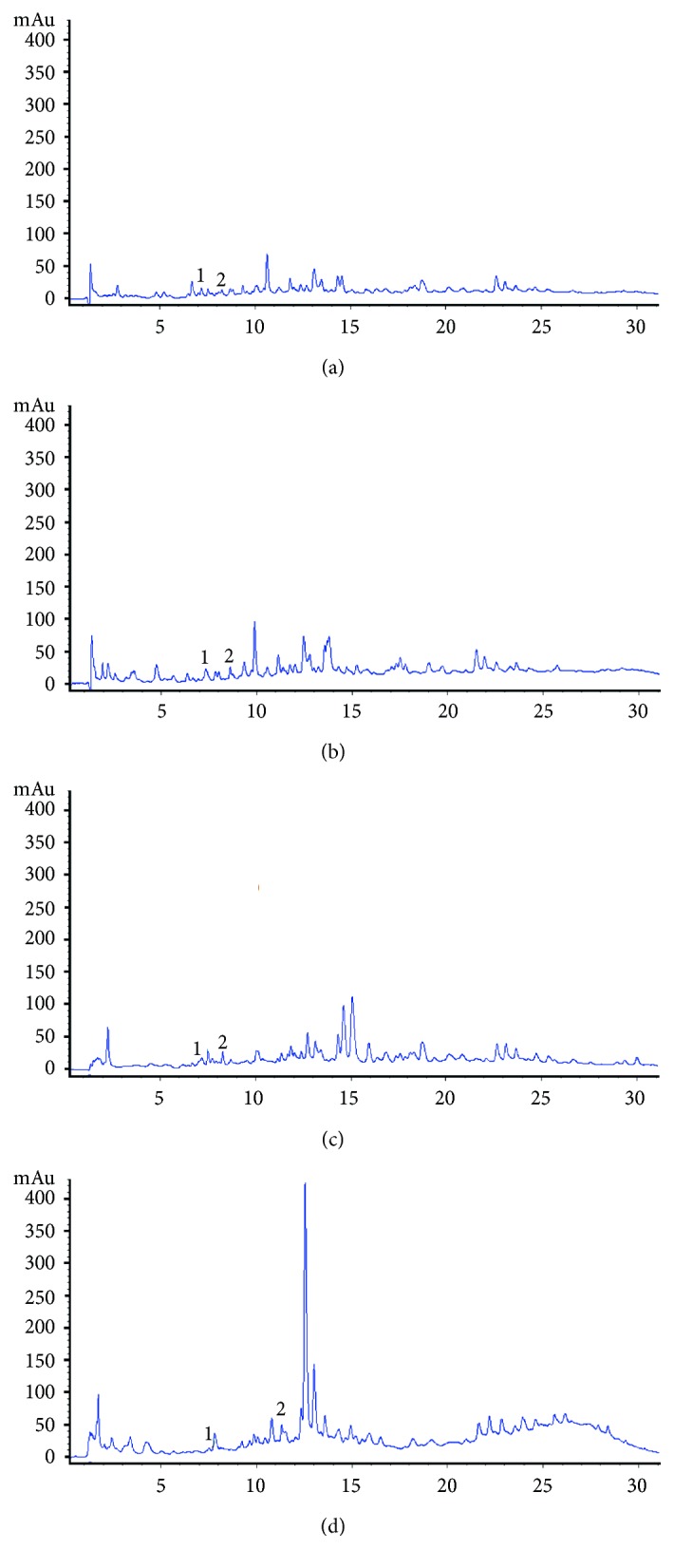
Chagalapoli (*Ardisia compressa*) HPLC Chromatograms at 280 nm: (a) filtered juice; (b) partially filtered juice; (c) filtered fermented beverage; (d) partially filtered fermented beverage.

**Figure 2:**
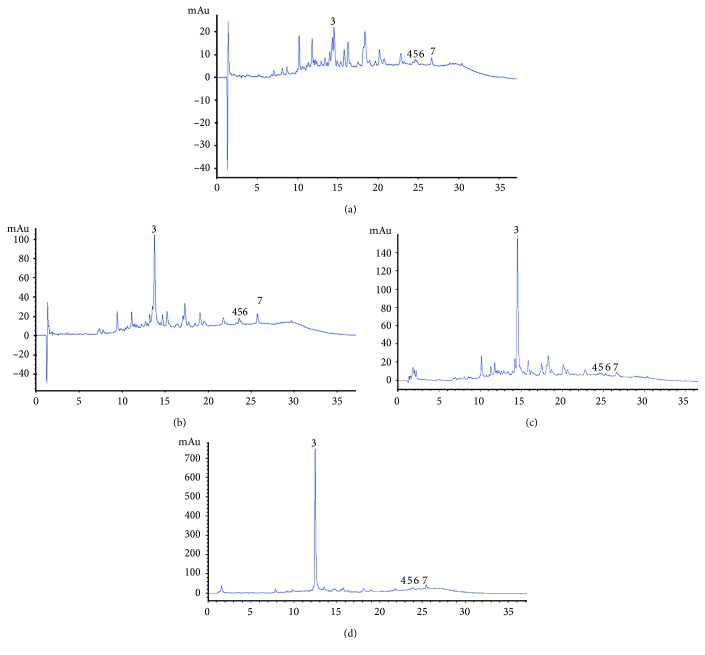
Chagalapoli (*Ardisia compressa*) HPLC chromatograms at 320 nm: (a) filtered juice; (b) partially filtered juice; (c) filtered fermented beverage; (d) partially filtered fermented beverage.

**Figure 3:**
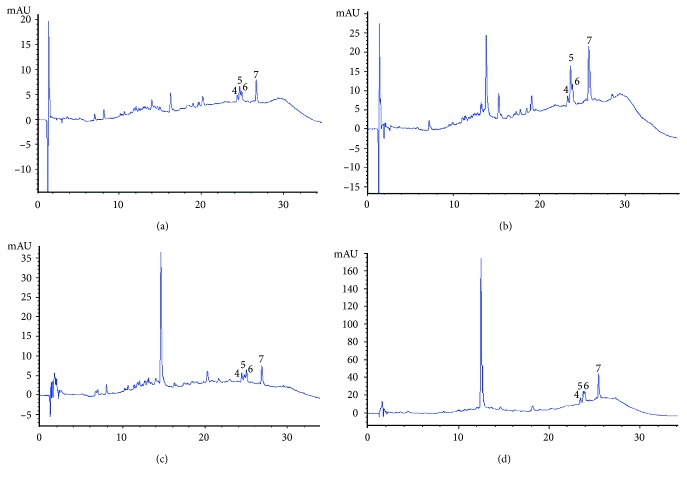
Chagalapoli (*Ardisia compressa*) HPLC chromatograms at 360 nm: (a) filtered juice; (b) partially filtered juice; (c) filtered fermented beverage; (d) partially filtered fermented beverage.

Total polyphenols are shown in Table 4. An increase in the total polyphenols was observed in both samples of Chagalapoli (*Ardisia compressa*) fermented beverages. At the beginning of the fermentation, the value was 1.07 ± 0.04 epichatechin mg/mL, with increases towards the end of fermentation with a final value of 1.47 ± 0.09 epichatechin mg/mL. The partially filtered chagalapoli sample initially presented 1.43 ± 0.1 epichatechin mg/mL and at the end of fermentation had increased to 2.86 ± 0.01 epichatechin mg/mL. Recent studies demonstrated the same behavior in fermentations in different grape varieties. In the fermentation process, several enzymes are excreted during the metabolism of the yeast, and these enzymes could act on the conjugated phenolic compounds to release free phenolic compounds, and thus change their composition [23].  Figure 1 show the compounds identified in *Ardisia compressa* samples at 280 nm wavelength. Peaks 1 and 2 correspond to catechin and epicatechin; catechin was found at 7.12 min up to 7.45 min, epicatechin was found at 8.2 min up to 8.54 min in the first chromatograms, while in the chromatogram of the partially filtered fermented beverage it was observed at 11.24 min.  Figure 2 shows the juices and fermented beverage chromatograms at 320 nm wavelength. In partially filtered fermented beverage, a major peak is shown, which was identified as chlorogenic at 12.48 min, 14.49 min and 13.73 min for filtered beverages and at 14.52 min in partially filtered juice.  Figure 3 shows compounds identified in *Ardisia compressa* beverages at 360 nm wavelength. Different derivatives of quercetin were identified between 24.65 min and 24.81 min for filtered beverages (juice and fermented beverages) and for partially filtered beverages they were identified at 23.23 min up to 23.92 min. Kaempferol was identified at 26.6 min in the filtered samples and in partially filtered samples it was found at 25.72 min and 24.48 min. Joaquín-Cruz et al. [4] identified 6 derivatives of quercetin with retention times of 18.61 min, 19.49 min, 19.92 min, 20.21 min, 22.98 min and 23.72 min. For the identification of kaempferol, retention times of 16.94 min and 17.24 min were obtained.

### 3.8. Sensory Evaluation

Fermented beverages from both batches were subjected to a sensory analysis to evaluate the degree of satisfaction of the final product, carried out with a panel of 32 untrained tasters older than 18 years.  Table 5 shows the results of acceptance of each sample, it was observed that the partially filtered beverage obtained a higher acceptance value in color and flavor parameters with a difference of 0.87 and 1.41, respectively. Aroma did not present a significant difference between samples; this means that there was not a preference for any of the two fermented beverages.

## 4. Conclusions

The results of the present study demonstrated that chagalapoli fruit has the potential to be used to produce fermented beverages. It was also revealed that chagalapoli fermented beverages represent a rich source of phenolic compounds and due to their phenolic composition they may be compared to other fruit fermented beverages. This beverage can be considered as a dry spirit since the content of reducing sugars was lower than 4 mg/mL. Based on the characteristics of the produced chagalapoli fermented beverages, it was concluded that there should be more extensive research with chagalapoli fruit due to its potential for development into a marketable beverage.

## Figures and Tables

**Table 1:** Proximal composition and physicochemical characteristics of chagalapoli (*Ardisia compressa*) fruit.

Components^a^	Value
Water (%)	80.52 ± 0.8
Ether extract (%)	0.55 ± 0.05
Ash (%)	0.66 ± 0.01
Protein (%)	8.58 ± 0.4
Crude fiber (%)	3.58 ± 0.005
Carbohydrates (%)	11.9
Water activity	0.97 ± 0.001
TSS^b^ (%)	17.7 ± 0.05
pH	4.2 ± 0.1

Results are expressed as mean ± SE (triplicate). ^a^Proximal components are expressed in fresh weight (FW). ^b^Total soluble solids (°Brix).

**Table 2:** Physicochemical properties of chagalapoli (*Ardisia compressa*) fruit filtered and partially filtered beverage during fermentation.

	Filtered fermented beverage					Partially filtered fermented beverage					
Days	0	1	2	3	4	0	1	2	3	4
pH	3.84 ± 0.04^a^	3.75 ± 0.06^ab^	3.65 ± 0.07^ab^	3.61 ± 0.09^b^	3.62 ± 0.09^b^	3.87 ± 0.2ª	3.74 ± 0.07ª	3.5 ± 0.1ª	3.51 ± 0.1ª	3.54 ± 0.1ª
SP. Gr^1^	1.072 ± 0.005^a^	1.048 ± 0.007^b^	1.028 ± 0.001^c^	1.009 ± 0.001^d^	1.004 ± 0^d^	1.074 ± 0^a^	1.044 ± 0.002^b^	1.030 ± 0.003^c^	1.006 ± 0.001^d^	1.004 ± 0.001^d^
TSS^2^ (%)	17.5 ± 0.1ª	11.16 ± 0.2^b^	7.09 ± 0.3^c^	2.34 ± 0.2^d^	1.04 ± 0^d^	17.9 ± 0^a^	11.03 ± 0.4^b^	7.74 ± 0.8^c^	1.7 ± 0.2^d^	1.2 ± 0.2^d^
TAC^3^ (g/L tartaric acid)	8.9 ± 0.1ª	8.6 ± 0.2ª	8.6 ± 0.08ª	8.6 ± 0.1ª	8.6 ± 0.08ª	8.9 ± 0.1ª	9.1 ± 0.2ª	9.3 ± 0.05ª	9.2 ± 0.1ª	9.2 ± 0.2^a^

Results are expressed as mean ± SE (triplicate). Values with same letter are not statistically different (*p* < 0.05).

^1^ Specific gravity.

^2^ Total soluble solids.

^3^ Titratable acidity.

**Table 3:** Chagalapoli (*Ardisia compressa*) juice and fermented beverage color analysis.

	*L* ^∗^	*a* ^∗^	*b* ^∗^	Chroma	Angle of hue (h°)
Filtered juice	27.50 ± 0.005^d^	8.87 ± 0.02ª	5.63 ± 0.02^c^	14.50± 0.01^d^	0.56 ± 0.002^d^
Filtered fermented beverage	30.34 ± 0.01^c^	8.42 ± 0.02^b^	9.07 ± 0.02^b^	17.50 ± 0.02ª	0.79 ± 0.002^c^
Partially filtered juice	30.75 ± 0.04^b^	7.15 ± 0.03^c^	9.44 ± 0.02ª	16.59 ± 0.03^b^	0.87 ± 0.002^b^
Partially filtered fermented beverage	31.09 ± 0.09ª	6.92 ± 0.06^d^	9.36 ± 0.09ª	16.28 ± 0.03^c^	0.87 ± 0.006ª

Results are expressed as mean ± SE (*n* = 5). Values with same letter are not statistically different (*p* < 0.05).

**Table 4:** Reducing sugar, total sugar and total polyphenol contents in filtered and partially filtered Chagalapoli (*Ardisia compressa*) fruit juice and filtered and partially filtered fermented beverages.

	Reducing sugar		Total sugar		Total polyphenols			
	mg (glucose)/mL		mg (glucose)/mL		Folin-Ciocalteu		HPLC-DAD	
					mg (GAE)/mL		mg (EPI)/mL	
	Juice	Fermented	Juice	Fermented	Juice	Fermented	Juice	Fermented
Filtered Chagalapoli	132.08 ± 1^a^	3.42 ± 0.3^b^	137.82 ± 0.9^a^	3.74 ± 0.1^b^	1.8 ± 0.03^a^	1.11 ± 0.02^b^	1.07 ± 0.04	1.47 ± 0.09
Partially filtered Chagalapoli	96.63 ± 2^a^	3.58 ± 0.3^b^	83.04 ± 0.6^a^	3.27 ± 0.03^b^	1.75 ± 0.1^a^	1.59 ± 0.19^b^	1.43 ± 0.1	2.86 ± 0.01

Results are expressed as mean ± SE (triplicate). Values with same letter are not statistically different (*p* < 0.05).

**Table 5:** Sensory analysis of chagalapoli (*Ardisia compressa*) fruit filtered and partially filtered fermented beverages.

	Filtered fermented beverage	Partially filtered fermented beverage
Color	3.47 ± 0.8^b^	4.34 ± 0.6ª
Aroma	3.69 ± 0.9ª	3.72 ± 1.2ª
Flavour	2.9 ± 0.1^b^	4.31 ± 0.8^a^

Results are expressed as mean ± SE (*n* = 32). Values with same letter are not statistically different (*p* < 0.05).
